# Detection of miRNA regulatory effect on triple negative breast cancer transcriptome

**DOI:** 10.1186/1471-2164-16-S6-S4

**Published:** 2015-06-01

**Authors:** Loredana Martignetti, Bruno Tesson, Anna Almeida, Andrei Zinovyev, Gordon C Tucker, Thierry Dubois, Emmanuel Barillot

**Affiliations:** 1 Institut Curie, Paris, France; 2 INSERM, U900, Paris, France; 3 Mines ParisTech, Fontainebleau, France; 4 Institut Curie, Department of Translational Research, Paris, France; 5 Institut Curie, Breast Cancer Biology Group, Paris, France; 6 Servier Research Center, Oncology, Croissy-sur-Seine, France

## Abstract

Identifying key microRNAs (miRNAs) contributing to the genesis and development of a particular disease is a focus of many recent studies. We introduce here a rank-based algorithm to detect miRNA regulatory activity in cancer-derived tissue samples which combines measurements of gene and miRNA expression levels and sequence-based target predictions. The method is designed to detect modest but coordinated changes in the expression of sequence-based predicted target genes. We applied our algorithm to a cohort of 129 tumour and healthy breast tissues and showed its effectiveness in identifying functional miRNAs possibly involved in the disease. These observations have been validated using an independent publicly available breast cancer dataset from The Cancer Genome Atlas. We focused on the triple negative breast cancer subtype to highlight potentially relevant miRNAs in this tumour subtype. For those miRNAs identified as potential regulators, we characterize the function of affected target genes by enrichment analysis. In the two independent datasets, the affected targets are not necessarily the same, but display similar enriched categories, including breast cancer related processes like cell substrate adherens junction, regulation of cell migration, nuclear pore complex and integrin pathway. The R script implementing our method together with the datasets used in the study can be downloaded here (http://bioinfo-out.curie.fr/projects/targetrunningsum).

## Background

MicroRNAs (miRNAs) are endogenous ~22 nucleotide RNA molecules that act as fundamental repressors of gene expression in many biological systems. In animals, they target mRNAs by recognizing and directly binding to multiple partially complementary sites preferentially located in the 3' untranslated regions (UTRs) of transcripts. Watson-Crick base-pairing to the 5' end of miRNAs, especially to the so-called 'seed' region that comprises nucleotides 2-7, is considered crucial for targeting [1,2], even if recently developed techniques for ligation and sequencing of miRNA-target RNA duplexes highlight widespread noncanonical seed interactions, containing bulged or mismatched nucleotides [3]. Although molecular mechanisms of miRNA action remain intensely debated [4], multiple studies revealed that mammalian miRNAs repress genes predominantly by destabilization of target mRNAs [5,6]. By computational and experimental approaches it was established that thousands of human protein-coding genes are regulated by miRNAs [7,8]. Given the wide scope of their targeting, miRNAs are considered as an additional layer of regulatory circuitry in the cell. Experimental observations suggest that miRNAs are regulators of development and cellular homeostasis through their control of diverse biological processes, from differentiation and proliferation to apoptosis [9]. Their role in regulating fundamental cell mechanisms suggests that they could be involved in cancer and indeed their expression is strongly deregulated in almost all human malignancies. Functional characterization of these aberrantly expressed microRNAs indicates that they might function as oncogenes and tumor suppressors [10,11].

Identifying key miRNAs contributing to the genesis and development of a particular disease is a focus of many recent studies. Statistical methodology for this task is not fully established due to the mild effect of miRNAs on the expression of their targets. A major source of information to infer the actual regulatory activity of miRNAs derives from high-throughput experimental data such as transcriptome profiles. The underlying assumption is that regulatory activity by miRNAs could be reflected by the expression changes of their target transcripts. For miRNAs that promote mRNA decay, there would be a negative correlation between miRNA and mRNA expression. Existing tools based on this assumption mainly rely on case-control mRNA profile experiments involving strong perturbations such as the knockout/knockdown/overexpression of one or few miRNAs [12-14]. In data originating from less controlled conditions, such as mRNA profiles of pathological tissue collected from patients, detecting miRNA-mediated target destabilization is more challenging due to presence of multiple cell types in samples, the activity of additional regulatory factors and complex RNA cross-regulation such as the miRNA sponge effect [15-17]. The recent study of a large cohort of breast tumour samples [18] suggests that miRNAs exert their effect by modulating mRNAs rather than acting as on-off switches. Other studies inferring miRNA regulation on tumour sample transcriptome exploit additional molecular information such as AGO2-PAR-CLIP binding-site data [19] or DNA copy number and promoter methylation at the mRNA gene locus [20].

**Figure 1:**
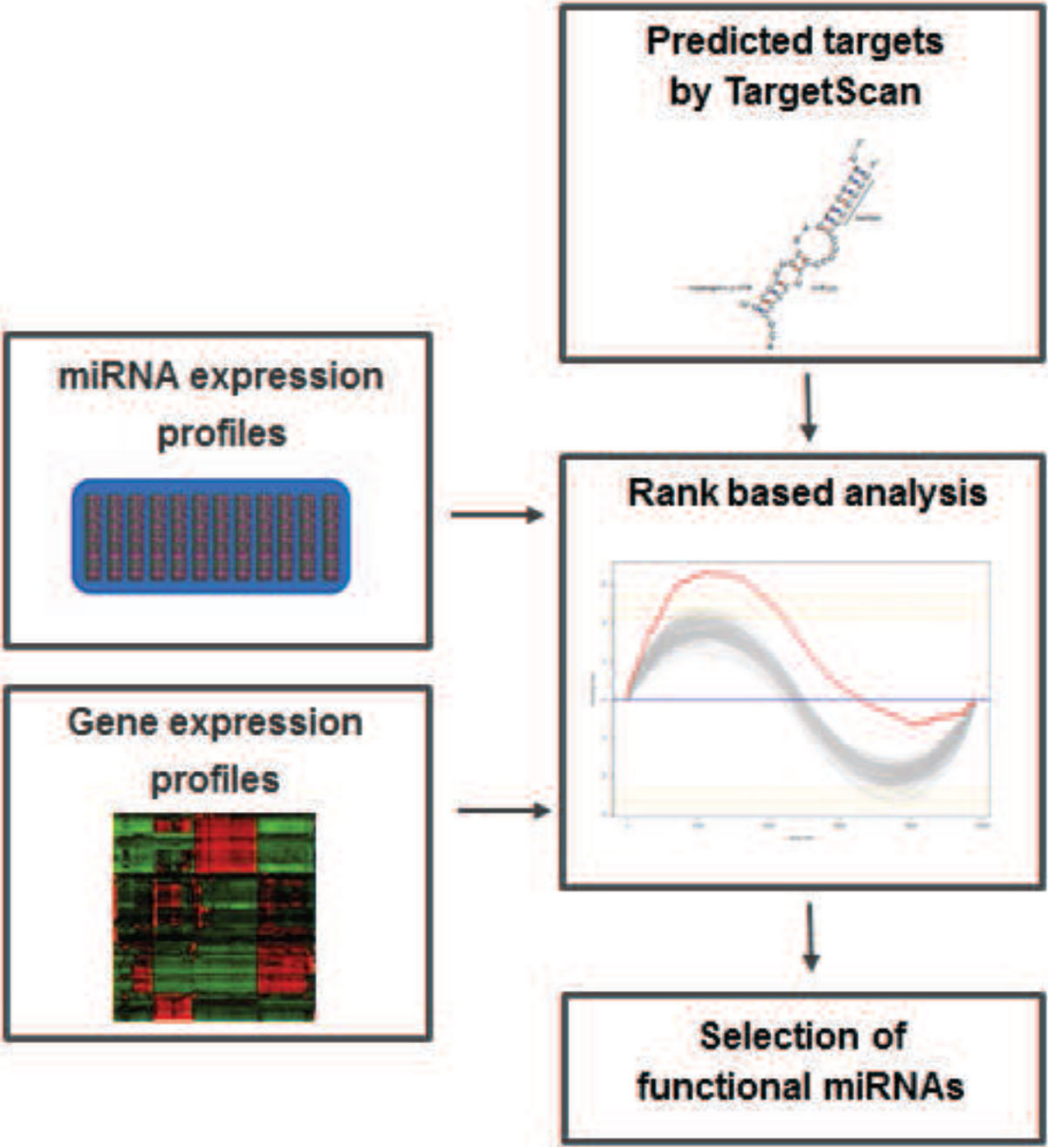
**Schematic summary of the pipeline**.

We introduce here a rank-based method to detect miRNA regulatory activity combining three sources of information, namely measurements of gene and miRNA expression levels from the same biological samples and sequence-based target predictions. Rank-based approaches such as Gene Set Enrichment Analysis (GSEA) [21] are designed to detect modest but coordinated changes in the expression of sets of functionally related genes. This is particularly suitable to infer miRNA regulatory effect from tissue expression profiles, in which this effect is subtle at the level of individual genes but affects a large number of genes. The original GSEA algorithm ranks all genes based on the correlation of their expression with a phenotype of interest and looks for predefined groups of functionally related genes that are enriched at either the top or bottom of the ranked list. We propose here a new scoring scheme in which the enrichment profile is based on both the correlation between gene and miRNA expression levels and the confidence of sequence-based target prediction. The defined enrichment score for a given miRNA is expected to be high if most of its predicted targets are at the top or at the bottom of the ranked list. The significance of the enrichment score is evaluated by a permutation procedure. As final result we obtain miRNAs showing a statistically significant enrichment score, which we consider as potential regulators in the analyzed conditions. The analysis pipeline is summarized in Figure 1. It has been implemented as a freely available R script (code available at http://bioinfo-out.curie.fr/projects/targetrunningsum).

We applied our method to elucidate the regulatory effect of miRNAs on the breast cancer transcriptome. Breast cancer is classified into various subtypes mainly based on the immunohistochemical staining of estrogen (ER), progesterone (PR) and HER-2 (ERBB2) receptors. The complex nature and heterogeneity of this disease, particularly with regard to gene expression profiles, make it difficult to detect the shaping effect of miRNAs on the transcriptome. We applied our algorithm to a breast carcinoma dataset including gene and miRNA expression from normal and breast tumour samples (which we refer to as Maire dataset [22,23]) and we show that it is able to identify miRNAs with statistically significant enrichment score. These results are then compared with those obtained on an independent dataset of normal and breast tumour samples from The Cancer Genome Atlas (TCGA) [24]. We further focus on the triple negative breast cancer (TNBC) to highlight miRNAs potentially relevant in this particular tumour subgroup. This subtype is intensively studied due to the lack of effective targeted therapies. We ran our algorithm including only samples characterized as triple-negative breast tumours and identified a set of miRNAs showing statistically significant signal in both Maire and TCGA datasets.

Finally, we investigated miRNAs identified as potential regulators to characterize the function of their targets. In the proposed algorithm, hundreds of genes account for the enrichment signal of a single miRNA and we expect a subset of them to participate in common cellular functions. We use multiple annotation databases to infer biological processes affected by the identified miRNAs. For those identified in both datasets, the corresponding sets of target genes were analyzed separately. We observe that even if the specific genes accounting for the enrichment of biological categories among miRNA target genes detected in the Maire dataset and in the TCGA datasets are not necessarily the same, they are associated to common cancer-related pathways

## Results

### Functional miRNAs in breast cancer

The analyzed dataset includes 129 tumour and healthy breast tissues for which both miRNA and mRNA expression profiles are available (see Material and Methods). In this study, we used sequence-based target sets obtained from TargetScan version 6.2 [8,25], a widely used algorithm which takes into account sequences that match the seed region of each miRNA and evaluate their conservation in several species. The confidence of target predictions is calculated as described in Methods. We include in our study 394 miRNAs for which both expression data and TargetScan predictions are available.

We identified 136 miRNAs as potential regulators with *FDR <*0.1 (results are reported in Additional File 1, Table S1). Among the top significant miRNAs, we found several ones that are known to function as oncomirs, such as the members of miR 17-92 cluster and its paralog cluster miR-106b-25, miR-15 and miR-16 [26].

**Figure 2:**
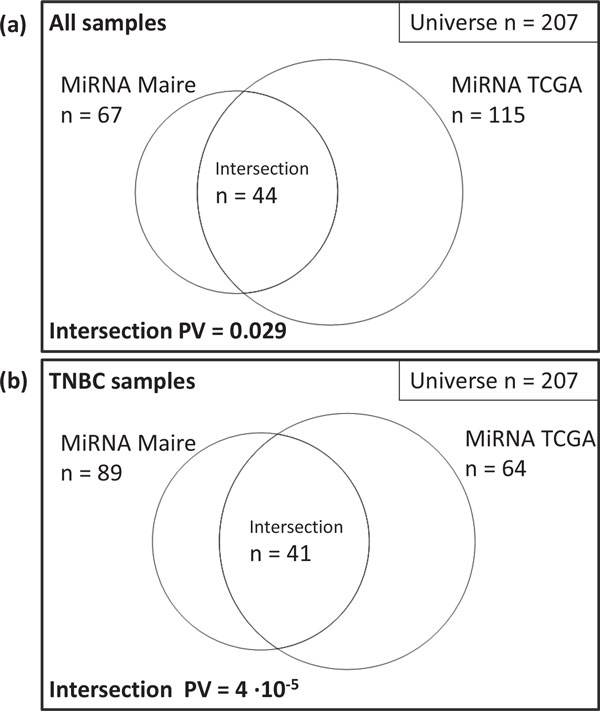
**Venn diagrams showing the overlap between results obtained in Maire cohort and TCGA when using all samples (a) and when restricting the analysis to the TNBC subtype samples (b)**.

To validate these results, we applied our algorithm to an independent dataset of 521 healthy and cancerous breast tissue samples from the TCGA project (see Material and Methods). This study includes 260 miRNA for which both expression data and TargetScan predictions are available. Of these, 142 miRNAs are identified as regulators with *FDR <*0.1. The intersection between results obtained in both datasets contains 44 miRNAs (see Figure 2a and Table 1) which corresponds to a statistically significant overlap (hypergeometric *p − value <*0.05).

**Table 1:** Common predicted miRNAs in Maire cohort and TCGA for the analysis including all samples.

MiRNA ID	ES MAIRE	PV Adj MAIRE	ES TCGA	PV Adj TCGA
hsa-miR-19b-3p	16.668	0.000	17.181	0.000
hsa-miR-19a-3p	16.050	0.000	17.233	0.000
hsa-miR-15b-5p	14.857	0.000	19.846	0.000
hsa-miR-17-5p	13.045	0.000	16.832	0.000
hsa-miR-106b-5p	12.655	0.000	15.708	0.000
hsa-miR-20a-5p	12.376	0.000	14.488	0.000
hsa-miR-26a-5p	12.045	0.000	6.143	0.000
hsa-miR-93-5p	11.830	0.000	12.905	0.000
hsa-miR-361-3p	11.819	0.000	12.931	0.000
hsa-miR-130b-3p	11.199	0.000	16.182	0.000
hsa-miR-18a-5p	10.669	0.000	11.522	0.000
hsa-miR-92a-3p	10.370	0.000	9.645	0.000
hsa-miR-16-5p	10.361	0.000	17.605	0.000
hsa-miR-20b-5p	10.307	0.000	7.941	0.000
hsa-miR-301a-3p	10.190	0.000	16.762	0.000
hsa-miR-222-3p	10.120	0.000	8.887	0.000
hsa-miR-331-3p	9.968	0.006	-14.024	0.000
hsa-miR-135b-5p	9.326	0.040	10.203	0.000
hsa-miR-107	9.113	0.021	11.283	0.000
hsa-miR-103a-3p	8.676	0.025	9.991	0.000
hsa-miR-141-3p	8.086	0.012	16.792	0.000
hsa-miR-29b-3p	7.733	0.000	15.204	0.000
hsa-miR-326	7.189	0.006	7.303	0.082
hsa-miR-193a-3p	7.008	0.000	7.302	0.000
hsa-miR-9-5p	6.933	0.025	7.196	0.045
hsa-miR-200c-3p	6.481	0.000	13.554	0.000
hsa-miR-150-5p	6.404	0.006	5.402	0.057
hsa-miR-193b-3p	6.038	0.054	9.071	0.028
hsa-miR-33a-5p	5.783	0.000	10.703	0.000
hsa-miR-205-5p	4.988	0.000	5.519	0.000
hsa-miR-92b-3p	4.873	0.038	4.876	0.000
hsa-miR-105-5p	3.729	0.091	7.058	0.000
hsa-miR-186-5p	3.680	0.006	7.578	0.000
hsa-miR-194-5p	3.443	0.006	4.639	0.003
hsa-miR-210	2.976	0.021	3.725	0.079
hsa-miR-339-3p	1.763	0.033	-2.476	0.072
hsa-miR-374a-5p	-2.640	0.000	4.346	0.039
hsa-miR-374b-5p	-3.187	0.000	5.686	0.000
hsa-miR-181c-5p	-6.795	0.000	-6.447	0.000
hsa-miR-582-5p	-6.816	0.000	7.163	0.006
hsa-miR-181d	-6.882	0.000	-5.433	0.000
hsa-miR-130a-3p	-7.903	0.015	8.103	0.034
hsa-miR-218-5p	-11.517	0.000	-9.091	0.065
hsa-miR-424-5p	-14.091	0.000	8.753	0.000

**Figure 3:**
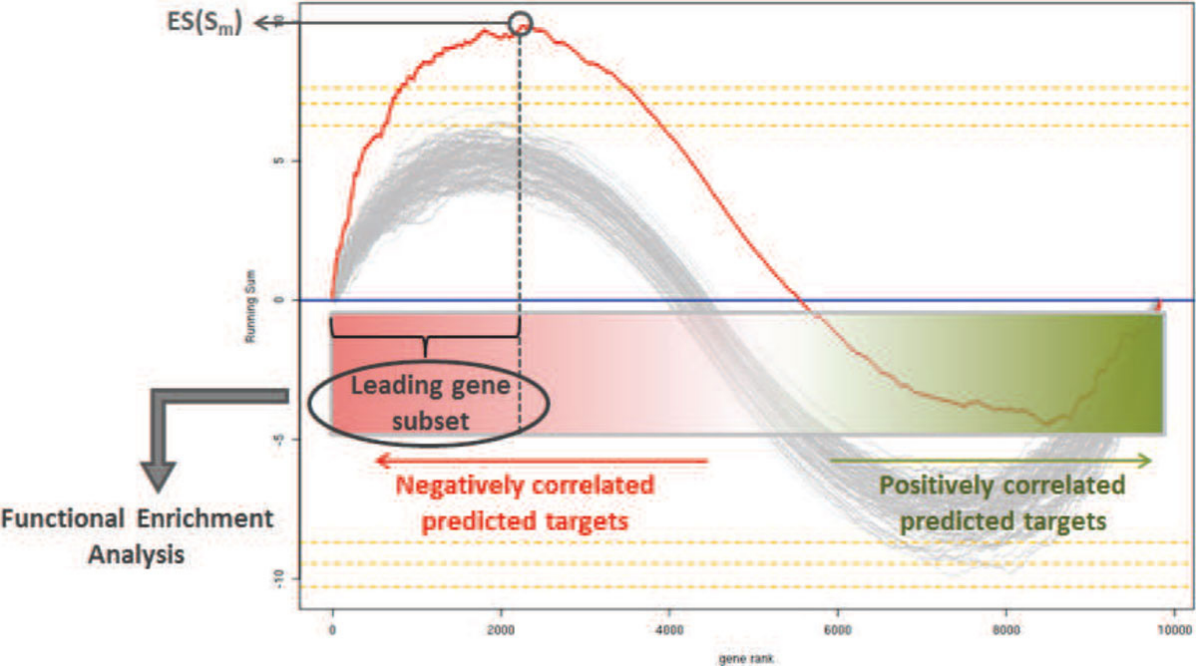
**An illustration of statistically significant enrichment score and selection of the leading-edge targets tested for functional enrichment**. For a given miRNA, the red line in the plot corresponds to the running sum profile obtained for the ranked list of predicted targets while the gray lines correspond to the profiles obtained when permuting the original list. Orange dashed lines indicate levels of significance with *p <*0.1, *p <*0.05 and *p <*0.01.

For a limited subset of 7 commonly identified miRNAs, the enrichment score obtained for the Maire dataset has opposite sign compared to that obtained for the TCGA dataset. In these cases, predicted targets are enriched among genes whose expression is positively correlated with the expression of the miRNA. The observation of miRNAs positively correlated with their predicted targets is in agreement with analogous integrated analysis of miRNA-mRNA correlation in tissue samples [27]. An interesting hypothesis suggests that this effect can be explained by common transcriptional regulation conferring robustness to gene expression program and ensuring tissue identity. Consistently, architectural features of the mammalian miRNA regulatory network reveal that the coordinated transcriptional regulation of a miRNA and its targets is an abundant motif in gene networks [28-30]. In our analysis, we consider both positive and negative correlation of predicted targets as a good evidence to infer miRNA regulation. We investigated whether the correlation sign of the miRNA expression with that of its targets is associated to a different proportion of correlated targets or a different correlation strength. For each significant miRNA we extracted the leading-edge subset of genes, corresponding to those genes in the set *S_m _*that appear in the ranked list before the point where the running sum achieves the ES (see Figure 3). This can be interpreted as the core of a gene set that accounts for the enrichment signal. We plotted the size of the leading-edge subset and the maximum correlation value as function of the correlation sign. Despite the size of the leading-edge subset and its maximum correlation value do not differ significantly according to the sign of the correlation, results show a trend toward stronger correlations of negatively correlated leading targets (Additional File 2, Figure S1).

### Triple negative breast cancer specific study

Of all breast tumours, TNBC is a very malignant subtype with poorly characterized molecular pathogenesis [31]. To elucidate the role of miRNA regulation in this specific cancer subtype, we applied our algorithm to the Maire dataset including only TNBC samples (n = 37). Similarly, we performed the analysis on the TCGA dataset selecting only TNBC samples (n = 82). Tumors were assigned to this subgroup according to ER, PR and HER2 negative status. Using TargetScan predictions, the algorithm identified 166 significant miRNAs in the Maire dataset (89 of them included in the common universe of miRNAs analyzed in both datasets) and 75 (64) in the TCGA dataset with *FDR <*0.1. The agreement between results obtained in the two datasets is highly significant (41 commonly identified miRNAs, hypergeometric *p − value <*10^*−*4^). Results are reported in Figure 2b and Table 2.

**Table 2:** Common predicted miRNAs in Maire cohort and TCGA for the TNBC specific analysis.

MiRNA ID	ES MAIRE	PV Adj MAIRE	ES TCGA	PV Adj TCGA
hsa-miR-361-3p	20.919	0.000	18.085	0.006
hsa-miR-29c-3p	13.774	0.000	10.705	0.000
hsa-miR-15b-5p	13.253	0.000	19.566	0.046
hsa-miR-423-5p	12.453	0.050	21.584	0.000
hsa-miR-27a-3p	11.658	0.000	13.731	0.000
hsa-miR-141-3p	11.144	0.000	15.045	0.000
hsa-miR-19a-3p	10.867	0.000	18.985	0.000
hsa-miR-17-5p	10.668	0.000	18.850	0.000
hsa-miR-29a-3p	10.612	0.000	11.034	0.000
hsa-miR-200c-3p	10.544	0.000	13.382	0.000
hsa-miR-93-5p	10.335	0.000	14.912	0.000
hsa-miR-107	10.263	0.019	12.125	0.034
hsa-miR-23a-3p	10.164	0.071	14.869	0.000
hsa-miR-19b-3p	9.860	0.000	19.694	0.000
hsa-miR-148a-3p	9.336	0.057	10.190	0.074
hsa-miR-20a-5p	9.117	0.000	18.901	0.000
hsa-miR-224-5p	9.020	0.000	6.678	0.006
hsa-miR-221-3p	8.173	0.000	5.772	0.019
hsa-let-7a-5p	7.996	0.027	7.920	0.006
hsa-miR-20b-5p	7.947	0.000	10.067	0.000
hsa-miR-30b-5p	7.538	0.027	8.141	0.000
hsa-miR-194-5p	7.528	0.000	5.670	0.034
hsa-miR-30a-5p	7.527	0.000	8.302	0.019
hsa-miR-429	7.443	0.000	11.493	0.000
hsa-miR-363-3p	7.183	0.007	8.978	0.000
hsa-miR-25-3p	7.011	0.000	13.278	0.000
hsa-miR-200a-3p	6.747	0.030	12.170	0.000
hsa-miR-222-3p	6.733	0.030	6.654	0.000
hsa-miR-106b-5p	6.726	0.014	16.857	0.000
hsa-miR-18a-5p	6.644	0.000	12.022	0.000
hsa-miR-155-5p	6.425	0.030	6.756	0.000
hsa-miR-200b-3p	6.383	0.000	11.561	0.000
hsa-miR-92a-3p	5.829	0.019	14.132	0.000
hsa-miR-192-5p	5.057	0.030	5.066	0.028
hsa-miR-576-5p	4.037	0.064	6.135	0.011
hsa-miR-127-3p	1.878	0.024	2.382	0.011
hsa-miR-374b-5p	-4.373	0.000	4.464	0.070
hsa-miR-139-5p	-6.743	0.000	-9.074	0.000
hsa-miR-218-5p	-8.715	0.000	-10.619	0.000
hsa-miR-26a-5p	-9.097	0.000	5.674	0.070
hsa-miR-130a-3p	-11.434	0.000	11.721	0.000

A group of 20 miRNAs is identified specifically in the TNBC study. Remarkably, some of these are already associated with aggressive breast cancer: miR-29a, miR-29c and miR-148a have been shown to be downregulated and associated to aberrant hypermethylation in basal-like cell line [32], miR-27a is involved in endothelial differentiation of breast cancer in a basal-like cell line [33] and in the MDA-MB-231 basal-like cell line [34] and miR-139-5p is described as a regulator of breast cancer cell motility and invasion [35].

### Identification of biological processes targeted by miRNAs

To assess the biological relevance of miRNAs identified as potential regulators, we investigated whether the genes that account for the enrichment signal of a given miRNA participate in the same cellular process or signalling pathway. The assumption that some miRNAs downregulate a group of genes participating in the same pathway is supported by multiple experimental studies [36-38]. Based on this hypothesis, for each significant miRNA we tested the leading-edge subset of genes for functional enrichment using curated annotation databases, such as Gene Ontology [39], KEGG [40], BioCarta [41], Reactome [42] and ACSN [43].

For miRNAs identified in both Maire and TCGA datasets, the corresponding subsets of leading-edge targets were analyzed separately. Interestingly, these subsets of genes display highly significant overlap and similar enriched categories, supporting the relevance of miRNA regulatory role in breast cancer. We report in Additional File 3, Table S2 the complete list of enriched categories with Bonferroni corrected p-values below 10*−*2. The most enriched categories include breast cancer related processes like cell substrate adherens junction, regulation of cell migration, nuclear pore complex and integrin pathway.

## Conclusions

High-throughput mRNA and miRNA expression data from large cohorts of normal and pathological tissue samples can be exploited to detect miRNA regulatory activity. The rank-based algorithm introduced here is able to detect miRNA-mediated target destabilization from normal and breast cancer expression profiles. Reproducible results were obtained in two independent datasets, providing a list of miRNAs potentially relevant in breast cancer. Moreover, the association of these miRNAs to cancer related processes is supported by functional enrichment of affected target genes.

The fact that a better overlap was obtained between Maire cohort and TCGA when restricting the analysis to the TNBC subtype compared to when using all samples may at first seem counterintuitive since using more samples should allow better power to detect correlations. However, this observation may be explained by the fact that when considering all breast cancer subtypes and healthy samples together, a larger part of the variation in the transcriptome data will arise from factors that are not directly linked to miRNA activity. For example, when putting together data from the luminal and non luminal subtypes, much of the variation will be associated with the status of the estrogen receptor pathway. Such variation can induce an important correlation structure in the data that may confuse the detection of the much subtler variation associated with miRNA regulation. The proposed algorithm can be considered a suitable tool to elucidate the regulatory role of miRNAs in physiological conditions.

## Methods

### Computational framework

The proposed algorithm requires as input genome-wide miRNA and gene expression data from the same biological samples and sequence-based predicted miRNA target sets. A three step procedure is applied to each miRNA *m*:

1. All genes are ranked according to the correlation between gene expression and the expression of miRNA *m*

2. The enrichment score defined in Equation (1) is computed for the sequence-based target set *S_m _*associated to the miRNA *m*

3. The significance of the enrichment score *ES*(*S_m_*) is evaluated by a permutation procedure estimating an empirical p-value *PV*(*ES*(*S_m_*))

The ranking scheme and the enrichment score definition are described as follows.

Let *G *= {*g*_1_*, g*_2_*, ...g_N _*} denote the list of all genes included in a genome-wide transcriptome experiment. For a given miRNA *m*, we sort this list according to the non-parametric (Spearman) correlation between gene expression and the expression of the miRNA *m *and get a ranked gene list *G_m _*= {*g*_*m*1_, *g*_*m*2_, ...*g*_*mN*_}. Given the sequence-based target set *S_m _*⊂ *G *associated with the miRNA *m *and the corresponding prediction confidence weights *W_m_*, we define the enrichment score as the running sum's maximal deviation from zero over all genes:

(1) $$ES\left(S_{m}\right)=\underset{1\leq i\leq N}{\text{max}}\left\{\overset{i}{\underset{j=1}{\sum }}\left(s_{mj}\cdot {\left|r_{mj}\right|}^{\alpha }-<s_{m}\cdot {\left|r_{m}\right|}^{\alpha }\right)\right\}\;with\;W_{m}=\left\{\begin{array}{cc} s_{mj} & \text{for}\;\;g_{mj}\in S_{m}\\ 0 & elsewhere \end{array}\right\}$$

where *r_mj _*is the correlation between the expression of gene *j *and the expression of miRNA *m, < s_m _· |r_m_|^α ^>*is the average of prediction confidence-weighted correlations for the set *S_m_*. The parameter *α *controls the contribution of the correlation *r_mj _*such that correlation values can contribute linearly (*α *= 1) or non-linearly in the running sum. Similar to the original GSEA algorithm, in this study, we set *α *= 1. According to this equation, the running sum is incremented by value (*s_mj _· |r_mj_|^α^− < s_m _· |r_m_|^α ^>*) when encountering a gene in *S_m _*and decreased by *< s_m _· |r_m_|^α ^>*when not.

The statistical significance of the enrichment score *ES*(*S_m_*) is assessed by a permutation based p-value. The enrichment score of the randomly shuffled list *G_m _*is computed for *N_p_*=1000 permutation rounds and the empirical null distribution of *ES*(*S_m_*) is generated. An empirical p-value *PV*(*ES*(*S_m_*)) is estimated for the positive and the negative region of the distribution by the proportion of permutations that result in larger *ES*(*S_m_*) than originally observed (or lower *ES*(*S_m_*) for the negative region). Once *PV*(*ES*(*S_m_*)) for a miRNA *m *is below a fixed threshold, the regulatory activity of miRNA *m *on the transcriptome is inferred. The *PV *threshold is set according to False Dicovery Rate (FDR) obtained by the Benjamini-Hochberg method [44]. In our study, the *PV *threshold was set according to *FDR <*0.1.

### Tissue collection

Healthy samples from mammary plastic surgery and tumor samples were obtained from patients treated at the hospital of Institut Curie (Biological Resource Center, Paris, France) as described previously [22,23]. Experiments were performed in agreement with the Bioethic Law No. 2004-800 and the Ethic Charter from the French National Institute of Cancer (INCa), and after approval of the ethics committee of our Institution. From the initial dataset, we retained only the samples for which both mRNA and miRNA data were available, numbering to 129, including 11 healthy breast tissue samples, 37 TNBC, 28 ER-/HER2+, 24 Luminal A and 29 Luminal B breast tumour samples as characterized by immunohistochemical staining.

### MiRNA expression data

Samples were hybridized on the Agilent miRNA microarray kit (V3). One hundred ng of total RNAs was hybridized to the microarrays according to the manufacturer's instructions. Hybridized microarrays were scanned with a DNA microarray scanner (Agilent G2565BA) and features were extracted using the Agilent Feature Extraction image analysis tool with default protocols and settings. Data were first transformed using the reverse hyperbolic sine function and quantile normalized. The data were then corrected for a hybridization batch effect using a linear model including the hybridization series as a fixed effect. Next, probes with negative intensity values in 95% or more of the arrays were discarded, leaving 516 miRNAs for analysis. When technical replicates for a sample were present, they were subsequently averaged. Two samples displayed an outlier behavior evident from Principal Component Analysis (data not shown) and were discarded from this analysis. The dataset can be downloaded here at the following address: http://bioinfo-out.curie.fr/projects/targetrunningsum.

### Gene expression data

For the protein-coding transcriptome, the data from Affymetrix U133plus2 arrays was processed as described [22]. In summary, we used the brainarray HGU133Plus2-Hs-Entrez version 13 custom chipset definition file [45], data were then normalized with GC-RMA [46], technical batch artefacts were corrected using a linear model, and genes with noise-level expression in 95% or more arrays were filtered out leaving 11543 probesets each corresponding to a unique Entrez Identifier.

### MiRNA and gene expression data from TCGA

We conducted our study on the publicly available data of tumour and healthy breast tissues from TCGA described in [24]. We selected 500 tumors and 21 tumor-adjacent normal breast tissue samples for which both mRNA and miRNA data were available. Of the 500 tumors, 82 were assigned to the TNBC subtype according to ER, PR and HER2 negative status. In this dataset mRNA expression levels were determined by Agilent custom 244K whole genome microarrays and miRNA abundance was measured by Illumina sequencing technology. Level 3 released data contain quality controlled and processed data done by Broad Institute's TCGA workgroup with expression call for genes per samples. Gene level expression data were normalized by using Robust Multi-array Average (RMA) and expression values were gene centered. MiRNA expression was given as read counts normalized to relative read frequency in each sample. Detected miRNAs were defined as having more than 10 reads in at least 10% of the samples, leaving 332 miRNAs for analysis.

### Target predictions by TargetScan

In the TargetScan algorithm, the prediction score of a miRNA binding site depends on the level of conservation and sequence context criteria such as the distance of the target from the 3'UTR end and the AU composition of the flanking area. For each miRNA, we take as prediction confidence weight of its targets the total context scores generated by the algorithm for 3'UTRs aggregating the binding site scores. By construction, the total context scores range between -1 and 0. When total context score for multiple 3'UTR isoforms are predicted, we take the total context score of the longest 3'UTR isoform.

## Competing interests

The authors declare that they have no competing interests.

## Authors' contributions

LM and BT designed the computational procedure and wrote the paper. TD, AA and GT were responsible for the experiments and the data collection. EB and AZ provided their expertise and supervised this study.

## Supplementary Material

Additional File 1 **Table S1 - MiRNAs identified in the Maire cohort for the analysis including all samples**. Click here for file

Additional File 2 **Figure S1 - Boxplot showing the number of the leading-edge targets (a) and the maximum correlation value (b) as function of the correlation sign between the expression of the miRNA and that of its targets**. Click here for file

Additional File 3 **Table S2 - Predicted biological processes targeted by miRNAs identified in both Maire and TCGA datasets**. The file contains one datasheet for the analysis including all samples and one datasheet for the TNBC specific results. Click here for file

## References

[B1] Brennecke J Stark A Russell RB Cohen SM Principles of microRNA-target recognition PLOS Biology 2005 3 3 e85 10.1371/journal.pbio.0030085 15723116 PMC1043860

[B2] Brodersen P Voinnet O Revisiting the principles of microRNA target recognition and mode of action Nature Reviews Molecular Cell Biology 2009 10 2 141 148 10.1038/nrm2619 19145236

[B3] Helwak A Kudla G Dudnakova T Tollervey D Mapping the human miRNA interactome by CLASH reveals frequent noncanonical binding Cell 2013 153 3 654 65 10.1016/j.cell.2013.03.043 23622248 PMC3650559

[B4] Morozova N Zinovyev A Nonne N Pritchard LL Gorban AN Harel-Bellan A Kinetic signatures of microRNA modes of action RNA 2012 18 9 1635 55 10.1261/rna.032284.112 22850425 PMC3425779

[B5] Selbach M Schwanh¨ausser B Thierfelder N Fang Z Khanin R Rajewsky N Widespread changes in protein synthesis induced by microRNAs Nature 2008 455 7209 58 63 10.1038/nature07228 18668040

[B6] Guo H Ingolia NT Weissman JS Bartel DP Mammalian microRNAs predominantly act to decrease target mRNA levels Nature 2010 466 7308 835 840 10.1038/nature09267 20703300 PMC2990499

[B7] Bartel DP MicroRNAs: genomics, biogenesis, mechanism, and function Cell 2004 116 2 281 297 10.1016/S0092-8674(04)00045-5 14744438

[B8] Friedman RC Farh KKH Burge CB Bartel DP Most mammalian mRNAs are conserved targets of microRNAs Genome Res 2009 19 92 105 18955434 10.1101/gr.082701.108 PMC2612969

[B9] Kim Dh Gru¨n D van Oudenaarden A Dampening of expression oscillations by synchronous regulation of a microRNA and its target Nat Genet 2013 45 11 1337 44 10.1038/ng.2763 24036951 PMC3812263

[B10] Schickel R Boyerinas B Park SM Peter ME MicroRNAs: key players in the immune system, differentiation, tumorigenesis and cell death Oncogene 2008 27 45 5959 5974 10.1038/onc.2008.274 18836476

[B11] Esquela-Kerscher A Slack FJ Oncomirs - microRNAs with a role in cancer Nat Rev Cancer 2006 6 4 259 269 10.1038/nrc1840 16557279

[B12] van Dongen S Abreu-Goodger C Enright AJ Detecting microRNA binding and siRNA off-target effects from expression data Nat Methods 2008 5 12 1023 5 10.1038/nmeth.1267 18978784 PMC2635553

[B13] Liang Z Zhou H He Z Zheng H Wu J mirAct: a web tool for evaluating microRNA activity based on gene expression data Nucleic Acids Res 2011 39 W139 44 10.1093/nar/gkr351 21596785 PMC3125759

[B14] Rasmussen SH Jacobsen A Krogh A cWords - systematic microRNA regulatory motif discovery from mRNA expression data Silence 2013 4 1 2 10.1186/1758-907X-4-2 23688306 PMC3682869

[B15] Sumazin P Yang X Chiu HS Chung WJ Iyer A Llobet-Navas D Rajbhandari P Bansal M Guarnieri P Silva J Califano A An extensive microRNA-mediated network of RNA-RNA interactions regulates established oncogenic pathways in glioblastoma Cell 2011 147 2 370 81 10.1016/j.cell.2011.09.041 22000015 PMC3214599

[B16] Hansen TB Kjems J Damgaard CK Circular RNA and miR-7 in cancer Cancer Res 2013 73 18 5609 12 10.1158/0008-5472.CAN-13-1568 24014594

[B17] Tay Y Rinn J Pandolfi PP The multilayered complexity of ceRNA crosstalk and competition Nature 2014 505 7483 344 52 10.1038/nature12986 24429633 PMC4113481

[B18] Dvinge H Git A Graf S Salmon-Divon M Curtis C Sottoriva A Zhao Y Hirst M Armisen J Miska EA Chin SF Provenzano E Turashvili G Green A Ellis I Aparicio S Caldas C The shaping and functional consequences of the microRNA landscape in breast cancer Nature 2013 497 7449 378 82 10.1038/nature12108 23644459

[B19] Farazi TA Ten Hoeve JJ Brown M Mihailovic A Horlings HM van de Vijver MJ Tuschl T Wessels LF Identification of distinct miRNA target regulation between breast cancer molecular subtypes using AGO2-PAR-CLIP and patient datasets Genome Biol 2014 7 1 15 R29 10.1186/gb-2014-15-1-r9 PMC4053773 24398324

[B20] Jacobsen A1 Silber J Harinath G Huse JT Schultz N Sander C Analysis of microRNA-target interactions across diverse cancer types Nat Struct Mol Biol 2013 20 11 1325 32 10.1038/nsmb.2678 24096364 PMC3982325

[B21] Subramanian A Tamayo P Mootha VK Mukherjee S Ebert BL Gillette MA Paulovich A Pomeroy SL Golub TR Lander ES Mesirov JP Gene set enrichment analysis: a knowledge-based approach for interpreting genome-wide expression profiles Proc Natl Acad Sci USA 2005 102 43 15545 50 10.1073/pnas.0506580102 16199517 PMC1239896

[B22] Maire V Baldeyron C Richardson M Tesson B Vincent-Salomon A Gravier E Marty-Prouvost B De Koning L Rigaill G Dumont A Gentien D Barillot E Roman-Roman S Depil S Cruzalegui F Pierré A Tucker GC Dubois T TTK/hMPS1 is an attractive therapeutic target for triple-negative breast cancer PLoS One 2013 8 5 e63712 10.1371/journal.pone.0063712 23700430 PMC3658982

[B23] Maire V Nemati F Richardson M Vincent-Salomon A Tesson B Rigaill G Gravier E Marty-Prouvost B De Koning L Lang G Gentien D Dumont A Barillot E Marangoni E Decaudin D Roman-Roman S Pierré A Cruzalegui F Depil S Tucker GC Dubois T Polo-like kinase 1: a potential therapeutic option in combination with conventional chemotherapy for the management of patients with triple-negative breast cancer Cancer Res 2013 73 2 813 23 10.1158/0008-5472.CAN-12-2633 23144294

[B24] Cancer Genome Atlas Network Comprehensive molecular portraits of human breast tumours Nature 2012 490 7418 61 70 10.1038/nature11412 23000897 PMC3465532

[B25] Grimson A Farh KK Johnston WK Garrett-Engele P Lim LP Bartel DP MicroRNA targeting specificity in mammals: determinants beyond seed pairing Mol Cell 2007 27 1 91 105 10.1016/j.molcel.2007.06.017 17612493 PMC3800283

[B26] Cho WC OncomiRs: the discovery and progress of microRNAs in cancers Mol Cancer 2007 25 6 60 17894887 10.1186/1476-4598-6-60 PMC2098778

[B27] Enerly E Steinfeld I Kleivi K Leivonen SK Aure MR Russnes HG Rønneberg JA Johnsen H Navon R Rødland E Makela R Naume B Perala M Kallioniemi O Kristensen VN Yakhini Z Børresen-Dale AL miRNA-mRNA integrated analysis reveals roles for miRNAs in primary breast tumors PLoS One 2011 6 2 e16915 10.1371/journal.pone.0016915 21364938 PMC3043070

[B28] Shalgi R Lieber D Oren M Pilpel Y Global and local architecture of the mammalian microRNA-transcription factor regulatory network PLoS Comput Biol 2007 3 7 e131 10.1371/journal.pcbi.0030131 17630826 PMC1914371

[B29] Re A Corá D Taverna D Caselle M Genome-wide survey of microRNA-transcription factor feed-forward regulatory circuits in human Mol Biosyst 2009 5 8 854 67 10.1039/b900177h 19603121 PMC2898627

[B30] Riba A Bosia C El Baroudi M Ollino L Caselle M A combination of transcriptional and microRNA regulation improves the stability of the relative concentrations of target genes PLoS Comput Biol 2014 10 2 e1003490 10.1371/journal.pcbi.1003490 24586138 PMC3937125

[B31] Toft DJ Cryns VL Minireview: Basal-like breast cancer: from molecular profiles to targeted therapies Mol Endocrinol 2011 25 2 199 211 10.1210/me.2010-0164 20861225 PMC3035993

[B32] Sandhu R Rivenbark AG Mackler RM Livasy CA Coleman WB Dysregulation of microRNA expression drives aberrant DNA hypermethylation in basal-like breast cancer Int J Oncol 2014 44 2 563 72 24297604 10.3892/ijo.2013.2197 PMC3898722

[B33] Tang W Yu F Yao H Cui X Jiao Y Lin L Chen J Yin D Song E Liu Q miR-27a regulates endothelial differentiation of breast cancer stem like cells Oncogene 2013 10 Jun 10.1038/onc.2013.214 23752185

[B34] Mertens-Talcott SU1 Chintharlapalli S Li X Safe S The oncogenic microRNA-27a targets genes that regulate specificity protein transcription factors and the G2-M checkpoint in MDA-MB-231 breast cancer cells Cancer Res 2007 15;67 22 11001 11 10.1158/0008-5472.CAN-07-2416 18006846

[B35] Krishnan K Steptoe AL Martin HC Pattabiraman DR Nones K Waddell N Mariasegaram M Simpson PT Lakhani SR Vlassov A Grimmond SM Cloonan N miR-139-5p is a regulator of metastatic pathways in breast cancer RNA 2013 19 12 1767 80 10.1261/rna.042143.113 24158791 PMC3884652

[B36] Lal A Navarro F Maher CA Maliszewski LE Yan N O'Day E Chowdhury D Dykxhoorn DM Tsai P Hofmann O Becker KG Gorospe M Hide W Lieberman J miR-24 inhibits cell proliferation by targeting E2F2, MYC, and other cell-cycle genes via binding to "seedless" 3'UTR microRNA recognition elements Mol Cell 2009 35 5 610 625 10.1016/j.molcel.2009.08.020 19748357 PMC2757794

[B37] Xu N Papagiannakopoulos T Pan G Thomson JA Kosik KS MicroRNA-145 regulates OCT4, SOX2, and KLF4 and represses pluripotency in human embryonic stem cells Cell 2009 137 4 647 658 10.1016/j.cell.2009.02.038 19409607

[B38] Li Z Hassan MQ Jafferji M Aqeilan RI Garzon R Croce CM van Wijnen AJ Stein JL Stein GS Lian JB Biological functions of miR-29b contribute to positive regulation of osteoblast differentiation J Biol Chem 2009 284 23 15676 15684 10.1074/jbc.M809787200 19342382 PMC2708864

[B39] Harris MA Clark J Ireland A Lomax J Ashburner M Foulger R Eilbeck K Lewis S Marshall B Mungall C Richter J Rubin GM Blake JA Bult C Dolan M Drabkin H Eppig JT Hill DP Ni L Ringwald M Balakrishnan R Cherry JM Christie KR Costanzo MC Dwight SS Engel S Fisk DG Hirschman JE Hong EL Nash RS Sethuraman A Theesfeld CL Botstein D Dolinski K Feierbach B Berardini T Mundodi S Rhee SY Apweiler R Barrell D Camon E Dimmer E Lee V Chisholm R Gaudet P Kibbe W Kishore R Schwarz EM Sternberg P Gwinn M Hannick L Wortman J Berriman M Wood V de la Cruz N Tonellato P Jaiswal P Seigfried T White R Consortium GO The Gene Ontology (GO) database and informatics resource Nucleic Acids Res 2004 32 Database D258 D261 14681407 10.1093/nar/gkh036 PMC308770

[B40] Kanehisa M Goto S Sato Y Kawashima M Furumichi M Tanabe M Data, information, knowledge and principle: back to metabolism in KEGG Nucleic Acids Res 2014 42 Database D199 205 24214961 10.1093/nar/gkt1076 PMC3965122

[B41] Nishimura D BioCarta Biotech Software Internet Report 2001 2 3 117 120 10.1089/152791601750294344

[B42] Croft D Mundo AF Haw R Milacic M Weiser J Wu G Caudy M Garapati P Gillespie M Kamdar MR Jassal B Jupe S Matthews L May B Palatnik S Rothfels K Shamovsky V Song H Williams M Birney E Hermjakob H Stein L D'Eustachio P The Reactome pathway knowledgebase Nucleic Acids Res 2014 42 Database 472 7 10.1093/nar/gkt1102 PMC3965010 24243840

[B43] http://acsn.curie.fr

[B44] Benjamini Y Hochberg Y Controlling the false discovery rate: a practical and powerful approach to multiple testing J R Statist Soc B 1995 57 1 289 300

[B45] Dai M Wang P Boyd AD Kostov G Athey B Jones EG Bunney WE Myers RM Speed TP Akil H Watson SJ Meng F Evolving gene/transcript definitions significantly alter the interpretation of GeneChip data Nucleic Acids Res 2005 33 20 e175 10.1093/nar/gni179 16284200 PMC1283542

[B46] Wu ZI RA Gentleman R Martinez-Murillo F Spencer F Model-based background adjustment for oligonucleotide expression arrays Journal of the American Statistical Association 2004 99 909 917 10.1198/016214504000000683

