# Supplementary figures and images for: Detection of miRNA regulatory effect on triple negative breast cancer transcriptome
Source: BMC Genomics. 2015 Jun 1;16(Suppl 6):S4. doi: 10.1186/1471-2164-16-S6-S4 (PMC4460783; doi:10.1186/1471-2164-16-S6-S4)

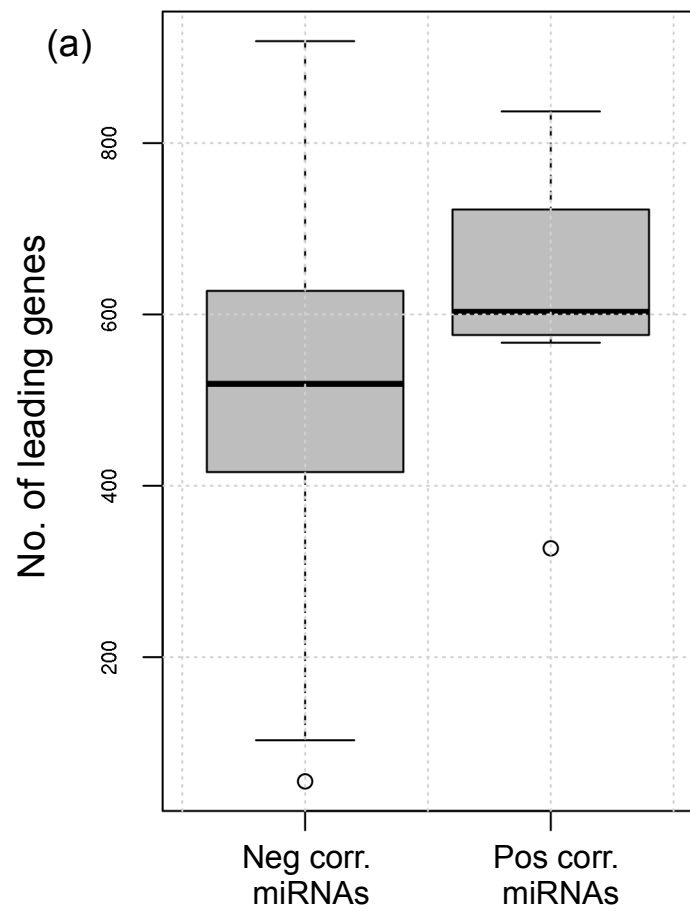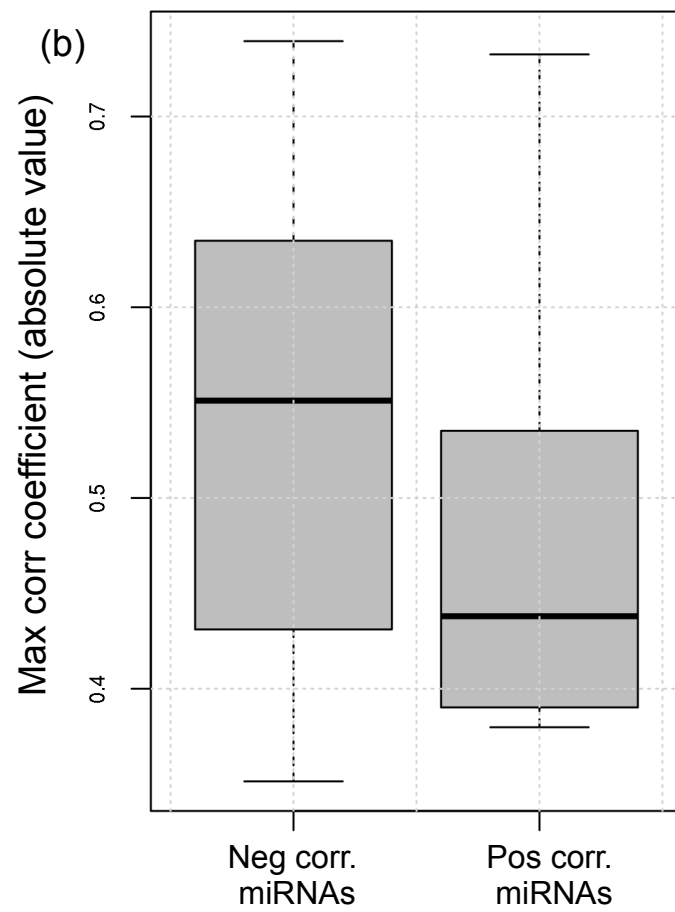

Supplement: Additional File 2 — Figure S1 - Boxplot showing the number of the leading-edge targets (a) and the maximum correlation value (b) as function of the correlation sign between the expression of the miRNA and that of its targets. [file 1471-2164-16-S6-S4-S2.pdf]
